# Management of red codes in the paediatric emergency room

**DOI:** 10.1186/1824-7288-40-S1-A60

**Published:** 2014-08-11

**Authors:** Paolo Biban, Simona Spada, Davide Silvagni, Monica Benedetti, Chiara Ghizzi

**Affiliations:** 1 Paediatric Emergency Room, Department of Paediatrics, Azienda Ospedaliera Universitaria Integrata Verona, Italy

## Background

Paediatric triage is a process to evaluate the conditions of children on their arrival in the Emergency Department. The goal of triage is to rapidly identify patients who are compromised or at risk of deterioration, assigning priorities of intervention and providing immediate assistance. The “red code” is attributed to any patient with potential or imminent risk of death, for whom evaluation and treatment should be immediately provided.

## Contents

Red codes are rarely assigned in the paediatric age. The main objective in these cases is to prevent death or brain damage in any patient with severe impairment of consciousness, breathing and/or circulation. Usually the patient is immediately received in a “shock room”, fully equipped with advanced monitoring systems, devices for airway and circulation management, drugs, manual defibrillator [1]. Initial actions are based on the ABCDE approach, i.e. Airway, Breathing, Circulation, Disability and Exposure. State of consciousness, airway’s patency, presence of respiratory arrest or insufficiency, arrhythmias or cardiac arrest must be quickly evaluated. In case of absence of vital signs, cardiopulmonary resuscitation (CPR) should be performed immediately by the attending healthcare providers, while seeking for additional help. CPR includes 100% oxygen, orotracheal intubation, chest compressions, early monitoring and defibrillation, intravascular or intraosseous access, drugs, fluids administration and temperature control. In case vital signs are present, every effort should be performed to avoid the progression toward cardiopulmonary arrest (CA). At the same time, the medical emergency team (MET) should be activated. The subsequent phase follows the algorithms for advanced management of the critically ill child, as recommended by international guidelines. Crucial steps are the recognition and treatment of potentially reversible causes of the CA, summarized in the “4 I”: hypoxia, hypovolemia, hypothermia, hypo-hyperkaliemia and the “4 T”: cardiac tamponade, thromboembolism, toxic, tension pneumothorax. Fast-ultrasound may be useful for quantitative diagnosis of some of these causes. After the initial stabilization the child should be transferred to other units for secondary care. A mention should be given to the presence of parents during invasive manoeuvres and CPR. In fact, a number of studies demonstrate that their presence during resuscitation efforts, may be helpful for them, particularly in case of poor outcome.

## Conclusions

Red codes to triage are attributed to children in cardiac arrest or imminent risk of death. Prompt management includes early recognition and treatment of cardiorespiratory insufficiency or cardiac arrest. Timely interventions and optimal quality of care may positively affect outcome.
